# Expansion of CD103^+^CD69^+^CD8^+^ cytotoxic liver tissue resident memory T cells and inflammatory monocytes in advanced biliary atresia

**DOI:** 10.3389/fimmu.2025.1567645

**Published:** 2025-06-18

**Authors:** Freya Sibbertsen, Regine J. Dress, Sören Alexander Weidemann, Katharina Möller, Uta Herden, Lutz Fischer, Kevin Paul, Jun Oh, Eva Tolosa, Sebastian Schulz-Jürgensen, Søren W. Gersting, Ania C. Muntau, Gábor A. Dunay

**Affiliations:** ^1^ University Children’s Research, University Medical Center Hamburg-Eppendorf, Hamburg, Germany; ^2^ Institute of Systems Immunology, Hamburg Center for Translational Immunology (HCTI), University Medical Center Hamburg-Eppendorf, Hamburg, Germany; ^3^ Department of Pathology, University Medical Center Hamburg-Eppendorf, Hamburg, Germany; ^4^ Department of Visceral Transplantation, University Medical Center Hamburg-Eppendorf, Hamburg, Germany; ^5^ Department of Pediatrics, University Children’s Hospital, University Medical Center Hamburg-Eppendorf, Hamburg, Germany; ^6^ Department of Immunology, University Medical Center Hamburg-Eppendorf, Hamburg, Germany; ^7^ German Center for Child and Adolescent Health (DZKJ), Hamburg, Germany; ^8^ University Medical Center Brandenburg Medical School (MHB), Klinikum Westbrandenburg, Brandenburg an der Havel, Germany; ^9^ Faculty of Health Sciences, Joint Faculty of the Brandenburg University of Technology Cottbus-Senftenberg, The Brandenburg Medical School Theodor Fontane and the University of Potsdam, Potsdam, Germany

**Keywords:** children, cirrhosis, Trm, hepatic monocytes, TNF-α

## Abstract

**Introduction:**

The pathogenesis of biliary atresia (BA) is unclear to date and no therapies targeting immune regulatory pathways exist. Here we characterized potent effector liver tissue resident memory CD8^+^ T cells (Trm) and monocytic cells in children with advanced BA and an age-matched control group to gain insight into BA pathogenesis and immunologic regulation.

**Methods:**

Liver explants from 18 children with biliary atresia and 10 with metabolic disease and normal histology were analyzed *ex vivo* by multicolor flow-cytometry and immunohistochemistry. Cytokines and cytotoxic mediators were quantified by intracellular staining and bead-based arrays in culture supernatant.

**Results:**

The frequency of CD103^+^CD69^+^CD8^+^ Trm cells and CD14^+^CD16^+^ monocytes was significantly higher in BA than in the control group. In BA, T cells showed elevated expression of CD103, CD69, CD39 and production of TNF-α and Granzyme-B *ex vivo*, which could be reproduced *in vitro* by allowing cell-contact with monocytes.

**Conclusions:**

Cytotoxic CD8^+^ Trm cells and intrahepatic monocytes might contribute to tissue destruction in BA. Therapies targeting Trm cells or the TNF-α signaling pathway could be explored to delay progression to cirrhosis in BA.

## 1 Introduction

Liver cirrhosis in children is a rare condition, and the immunologic mechanisms contributing to or complicating liver cirrhosis in children are poorly understood (1). The spectrum of liver diseases leading to cirrhosis in very young children is completely different from the adult population. Among the main causes of pediatric end-stage liver disease necessitating transplantation is biliary atresia, a disease leading to obliteration of the biliary tree beginning already during gestation (2). The operative Kasai portoenterostomy remains the only option to delay the progression of liver cirrhosis. However, most patients still require a liver transplantation very early in life, the only curative treatment for biliary atresia (2, 3).

Tissue resident memory T (Trm) cells are present in several organs, including the liver, and are defined in humans most typically by the expression of CD69 and/or CD103 (4). Trm cells may be anergic at steady-state but acquire cytotoxic potential upon antigenic rechallenge in healthy and diseased liver (5–7). In several infection models, hepatic CD8^+^ Trm cells showed superior memory recall responses compared to circulating effector cells, and Trm cells may also have a role in maintenance of tissue homeostasis, indicating their possible dichotomic function (7–9). Literature also suggests that Trm cells might contribute to liver damage due to their cytotoxicity (10, 11). Experimental studies in mice suggest that T cells especially of Th1-type polarization (12, 13) as well as IL-17 from γδ T cells (14) may have a pathogenic role in BA. Data on the pathogenic role of T cells from human pediatric cohorts is scarce because of low availability of these samples (15–17). To our knowledge, only one published work specifically analyzed Trm cells in primary liver samples from six children with BA and ten control children, showing their increased abundance compared to other, anti-fibrogenic T effector cell types (18). In this study, we investigated intrahepatic cellular immune phenotypes in histologically normal pediatric livers and cirrhotic livers of young children with biliary atresia, with the goal of elucidating the possible pathological role of CD8^+^ Trm cells in the development of pediatric liver cirrhosis.

We found increased frequency and cytotoxicity of CD8^+^ Trm cells in biliary atresia associated pediatric liver cirrhosis compared to the control group and describe how the interaction with tissue monocytes may promote this phenotype.

## 2 Materials and methods

### 2.1 Study approval

The study was approved by the local ethical committee of the Hamburg Medical Association (PV7150). The study was performed in accordance with the Declaration of Helsinki. Study participants were recruited among children listed for liver transplantation at the University Medical Center Hamburg-Eppendorf. The current study included children under 6 years of age and with a liver histology indicating advanced cirrhosis associated with progressed biliary atresia or a histologically normal liver with a metabolic condition undergoing liver transplantation. Children with intermediate histological findings were excluded from the study. Legal guardians provided written informed consent to participate in the study (**Supplementary Figure 1**). *In vitro* experiments were carried out with cells from buffy coats, obtained at the Department of Transfusion Medicine at the University Medical Center Hamburg-Eppendorf from adult blood donors, who provided their written informed consent.

### 2.2 Processing of liver samples

Liver explants were directly obtained from the operating room and processed immediately. Central as well as peripheral areas spanning multiple lobes were selected at random for sampling. Intrahepatic lymphocytes were isolated without enzymatic digestion by purely mechanical dissociation with the GentleMACs OctoDissociator (Miltenyi Biotec) using a program recommended by the manufacturer for processing cirrhotic liver tissue. The obtained cell suspension was filtered through a 70µm filter and carefully washed. Further separation was conducted by repeated centrifugations at low speed (40 x g, 4 minutes, room temperature). The supernatant, containing the desired liver mononuclear cell fractions was used for this study. This centrifugation step was repeated until no visible pellet was left. The mononuclear cell fraction was pelleted by centrifugation at 450 x g, 10 minutes at room temperature. Erythrocytes were lysed using RBC- lysis buffer (Thermo Fisher Scientific GmbH).

Samples of native liver tissue were cryopreserved in liquid nitrogen and stored at -196°C.

### 2.3 *In vitro* monocyte – CD8^+^ T cell interactions

Magnetic negative cell enrichment of CD8+ T cells and monocytes from buffy coats of adult healthy blood donors was performed using EasySep™ kits from STEMCELL Technologies according to the manufacturer’s instructions. Adult blood donors were used due to the limiting quantity of blood that can safely be obtained from children. CD14^+^ monocytes were cultured and differentiated in Rosewell Park Memorial Institute culture medium (RPMI, Gibco) with 10% fetal bovine serum (FBS, Capricorn Scientific) and 10ng/ml granulocyte-monocyte colony stimulating factor (GM-CSF, PreProtec) prior to the experiments, until they showed an elongated morphology, between 7–10 days. Cryopreserved autologous PBMCs were thawed in time to isolate CD8+ T cells. Purified CD8+ T cells and monocytes were cultured in RPMI + 10% FBS, 20 U/ml Interleukin-2 (IL-2, R&D), and 10 ng/ml GM-CSF, as well as Interleukin -15–50 ng/ml (IL-15, R&D), with the addition of TGF-β1–50 ng/ml (R&D) on day three at 37°C in a humidified atmosphere containing 5% CO_2_ (7). 10^6^ isolated CD8^+^ T cells were cultured alone or cocultured with 10^5^ autologous monocytes with or without a transwell-chamber with 0.4 µm pores (Corning, Sigma-Aldrich) to prevent or allow physical monocyte-T-cell contact. Cell-free supernatant was collected on day 7 for analysis and stored at -20°C.

### 2.4 Flow cytometry

Cryopreserved aliquots of cell suspensions were incubated for one minute in a 37°C water bath and subsequently thawed by gentle pipetting in 50 ml of pre-warmed RPMI, washed once with PBS, and stained with Near-IR Dead Cell Stain Kit (Invitrogen) incubated for 15 minutes in the dark at room temperature. Subsequently, cells were stained without intermediate wash with a prepared cocktail of fluorescently labeled antibodies for 20 minutes at room temperature (**Supplementary Table 1**) and then washed with PBS. Cells were kept at 4°C in PBS until analysis. For cell culture experiments, cells were harvested on the given day and washed once with PBS and the staining procedure was carried out as described above. Flow cytometry was performed using a BD FACS Symphony A3 cytometer or at a Cytek^®^ Aurora spectral flow cytometer (Cytek Biosciences) (after fixation of cells in 1% paraformaldehyde (PFA) for one hour at 4°C) or a BD AriaFusion in a biological safety cabinet at the Flow Cytometry Core Facility of the University Medical Center Hamburg-Eppendorf. Staining consistency was ensured using Rainbow beads (BD Sphero). For compensation controls, the Anti-Mouse or Anti-Rat Ig, κ/Negative Control Compensation Particles Set (BD Biosciences) was used for fluorescently labeled antibodies, and ArC™ Amine Reactive Compensation Beads (Invitrogen) were used for compensation of the Dead Cell Stain Kit (Life).

### 2.5 Intracellular staining and stimulation

10^6^ cells each were stimulated by adding 50 ng/ml phorbol myristate acetate (PMA) and 1 µg/ml ionomycin (both Sigma-Aldrich) in 5x10^6^ cells per milliliter RPMI with 10% FBS or left unstimulated in culture medium only. A Golgi block was added immediately using 1 mg/ml brefeldin-A (Sigma-Aldrich) and 2mM monensin (BioLegend). Cells were incubated for 6 hours at 37°C in a humidified atmosphere containing 5% CO_2_. Staining of surface markers was carried out as described above. For intracellular staining, permeabilization was carried out using the FOXP3 Fixation/Permeabilization Buffer Set (eBioscience) according to the manufacturer’s instructions with a slight adaptation, whereby cells were incubated with fluorescently labeled antibodies for intracellular targets for 40 minutes at 4°C in the dark. Cells were stored in PBS at 4°C until analysis.

### 2.6 LEGENDplex™

Detection of cytokines and cytotoxic mediators in the cell culture supernatant of stimulated cells was performed using LEGENDplex™ Human IL-15, Human Free Active TGF-β1 and Human CD8/NK Panel (13-plex) suitable for detection of IL-2, IL-4, IL-10, IL-6, IL-17A, TNF-α, sFas, sFasL, IFN-γ, Granzyme A, Granzyme B, Perforin, Granulysin, according to the manufacturer’s instructions (BioLegend). Quantification was carried out using the provided online LEGENDplex™ Data Analysis Software. Assays were performed in duplicates and mean values of each sample were used for further analysis. A freshly prepared standard ensured accurate quantification of the concentration of each target.

### 2.7 Immunohistochemistry

Freshly cut 4µm serial sections were deparaffinized, rehydrated and exposed to heat-induced antigen retrieval. For CD14 for 5 minutes in an autoclave at 121°C at pH 9.0 in Tris–EDTA-Citrate buffer. For CD8–15 min at 98°C in pH9 DAKO target retrieval Solution (S2367) using a DAKO PT-LINK device and then transferred to a DAKO Link 48 autostainer device. The autostainer protocol includes peroxidase blocking for 5 minutes (DAKO, Envision Flex-Kit 8002) and subsequent incubations of the primary antibody (CD8: DAKO, mouse monoclonal antibody, Clone C8/144B, ready to use; CD14: Cell Marque, rabbit monoclonal antibody, Clone EPR3653, ready to use) for 20 minutes at room temperature, Flex HRP (DAKO EnVision Flex-Kit 8002) for 20 minutes, DAB-Chromogen (DAKO EnVision Fley-Kit 8002) for 10 min as well as a final incubation with Hämatoxylin (DAKO K8008) for 5 minutes.

### 2.8 Quality control, bioinformatic analysis and visualization

Prior to the analyses, extensive quality control and sample cleanup was performed using FlowJo plugin FlowAI (19). CD45^+^ live cells passing quality control were used for bioinformatic analysis and visualization. Samples with less than 10.000 alive cells were excluded from further analysis. FlowJo plugins TriMap and PhenoGraph were used for visualization and clustering. TriMap, FlowAI and PhenoGraph were used with default settings, except for k, which was set to k=20 to identify smaller cell populations. BUV395-CD45 and APC-Cy7-LiveDead were excluded from the bioinformatic analysis after pre-gating on single, live, CD45^+^ events.

### 2.9 Statistical analysis

Data analysis was performed with GraphPad Prism 8 (GraphPad Software Inc). Two groups were compared using Student’s t-Test (unpaired, 2-tailed). Three groups were compared using one-way ANOVA and Tukey´s multiple comparisons test. Categorical parameters were compared using Fisher’s exact test. Outlier were identified using Grubbs´s Test with Alpha=0.05. Data are presented as mean ± SD. Results were considered significant at p < 0.05. All error bars represent SD.

The power analysis for our *in vitro* experiment indicates that a sample size of 7 per group is sufficient by a statistical power of 0.8, and we have utilized more than 10 samples per group. The R2 value for this analysis is 0.78, indicating a high level of explanatory power.

For the *ex vivo* data, the power analysis demonstrates that the observed effects on tissue resident memory T (Trm) cells and CD16^+^ intermediate monocytes have a power exceeding 0.99 for our collected sample size (n=10 and 18). This underscores the reliability of our *ex vivo* analysis and provides a strong basis for the interpretation of the observed effects in these specific cell populations.

## 3 Results

### 3.1 Children with biliary atresia exhibit features of advanced liver cirrhosis accompanied by inflammation

The study population consisted of 28 pediatric participants undergoing liver transplantation. The biliary atresia (BA) group included 18 children. Pathology reports were in line with the clinical diagnosis and indicated an advanced cirrhosis in all the liver explants. The control group included ten patients transplanted with inborn errors of metabolism including maple syrup urine disease (MSUD), ornithine carbamoyl transferase (OTC) deficiency, carbamoyl phosphate synthetase (CPS) 1 deficiency, citrullinemia type 1, argininosuccinic aciduria, glycogenosis type 1A and type 1B or factor V deficiency (**Table 1**), all of whom showed no histological signs of inflammation and cirrhosis in the pathology reports.

**Table 1:** Patient history and diagnosis.

Clinical data	n	Cirrhosis	Mutation in:
Biliary atresia	18	yes	n/a
CPS 1 deficiency	1	no	Carbamoyl phosphate synthetase 1
Citrullinemia type 1	1	no	Arginosuccinate synthetase
Argininosuccinic aciduria	2	no	Argininosuccinic acid lyase
OTC deficiency	2	no	Ornithine transcarbamylase
Maple syrup urine disease	1	no	Branched-chain α-ketoacid dehydrogenase complex
Glycogenosis type 1A	1	no	Glucose-6-phosphatase
Glycogenosis type 1B	1	no	SLC37A4
Factor V deficiency	1	no	Factor V

CPS, carbamoyl phosphate synthetase; OTC, ornithine transcarbamylase.

Clinical characteristics including main laboratory findings in the two groups (control vs BA) are shown in **Table 2**. Children in the BA group showed features of advanced liver cirrhosis (cholestasis, mild- to moderately increased liver transaminases, decreased synthetic function) and portal hypertension (thrombocytopenia), as well as signs of inflammation (leukocytosis and increased CRP), whereas children in the control group had normal laboratory findings.

**Table 2:** Patient characteristics and clinical parameters.

Parameter	P-value	Mean ± SD of control group	Mean ± SD of biliary atresia
Age (in months)	0.1530	20.2 ± 19.7	9.9 ± 12.1
gender (f) in %	0.1000	90	55
Hb g/dL	0.0161	11.3 ± 1.7	9.78 ± 1.37
Thrombocytes G/L	0.0002	466.2 ± 249.3	177.12 ± 98.09
Leucocytes G/L	0.0509	9.1 ± 4.2	13.48 ± 5.94
Lymphocytes G/L	0.6195	5.4 ± 2.91	4.85 ± 2.38
Neutrophiles G/L	0.0279	2.8 ± 2.31	6.21 ± 3.91
Ammonia µmol/l	0.0427	47 ± 16.4	85 ± 38.19
Albumin g/L	<0,0001	38.9 ± 4.8	24.9 ± 4.68
Bilirubin total	<0,0001	0.3 ± 0.2	19.15 ± 9.04
Bilirubin conjugated			14.76 ± 9.25
AST U/L	0.0030	55.6 ± 30.4	409.93 ± 333.14
ALT U/L	0.0176	59.6 ± 81.4	226.07 ± 192.21
GGT U/L	0.0655	28.6 ± 16.9	230 ± 324.01
ALP U/L	0.0053	257 ± 76.8	502.33 ± 199.6
CRP (mg/l)		<4	30.6 ± 51.24
Quick %	<0.0001	88.6 ± 23	49.77 ± 17.79
INR	0.0078	1.1 ± 0.2	1.55 ± 0.49
APTT Sec	0.0132	34.8 ± 6.3	49.13 ± 16.08
ATIII %	0.0001	107.9 ± 18.4	62 ± 22.08
Fibrinogen acc. Claus g/L	0.2444	2.1 ± 1.3	1.53 ± 0.69
Fibrinogen g/L	0.5201	3 ± 0.6	2.72 ± 0.72

BA, biliary atresia; Hb, hemoglobin; AST, Aspartate aminotransferase; ALT, Alanine aminotransferase; GGT, Gamma-glutamyltransferase; ALP, Alkaline phosphatase; INR, International normalized ratio; APTT, Activated partial thromboplastin time; ATIII, Antithrombin III.

### 3.2 CD103^+^CD69^+^CD8^+^ Trm cells and CD16^+^CD14^+^ intermediate monocytes constitute a hallmark of advanced biliary atresia

To obtain a detailed characterization of pediatric intrahepatic lymphocyte and myeloid populations, we performed deep immune phenotyping by flow cytometry on single cell suspensions isolated from pediatric liver explants in the biliary atresia (BA) and control groups.

**Figure 1:**
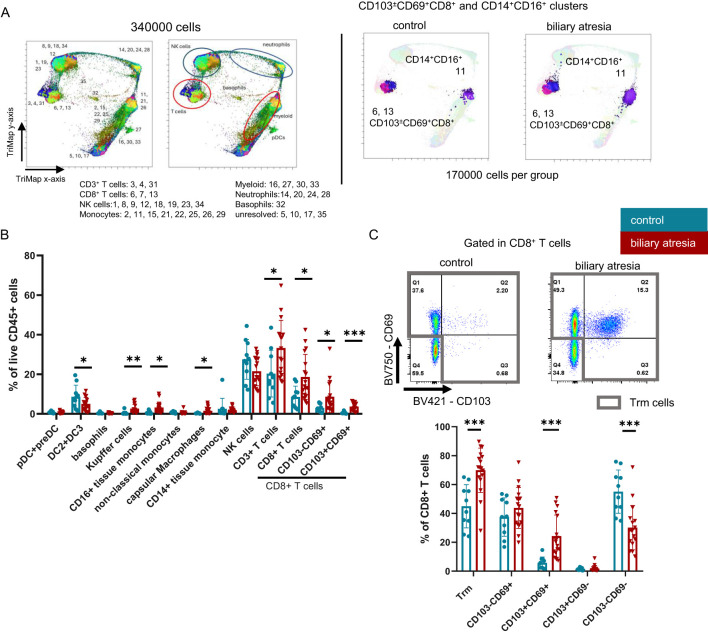
Frequencies of CD103^+^CD69^+^CD8^+^ Trm cells and intermediate CD14^+^CD16^+^ monocytes are increased in biliary atresia. **(A)** Equal number of each patient per group were concatenated to a final number of 170000 CD45^+^live cells per group. 170000 CD45^+^ live cells from both groups were concatenated, visualized using the FlowJo Pugin TriMap using default settings and clustered using PhenoGraph, with k set to 20. Each cluster was annotated based on the expression of linage markers. The population to which each cluster belongs is annotated in the figure and the annotation of each respective cluster is shown in **Supplementary Figure 3A**. CD103 ^±^ CD69^+^CD8^+^ and CD14^+^CD16^low/+^ clusters are shown highlighted in their respective color (control group: n=10; biliary atresia: n=17). **(B)** Populations identified with manual gating in the CD45^+^ live population (control group: n=10; biliary atresia: n=17). **(C)** Frequencies of CD8^+^ Trm cells and subpopulations in CD8^+^ T cells (control group: 10; biliary atresia: n=17). Data are presented as mean ± SD. *p<0.05; **p<0.01; ***p<0.001. **(B, C)** Unpaired t-test.

Trm cells frequently are characterized by the expression of tissue retention markers, CD69 alone or in combination with CD103. Both, CD103^-^CD69^+^CD8^+^ (single positive, SP) and CD103^+^CD69^+^CD8^+^ (double positive, DP) Trm cells were increased in BA. Statistical analysis of both the unbiased clustering tool (PhenoGraph) and manual gating of intrahepatic lymphocytes revealed a significant increase in CD8^+^ Trm cells, more specifically an increase in both SP and DP Trm cells in BA (cluster 6 and cluster 13, respectively) (**Figures 1A, B**, **Supplementary Figures 2, 3A**). In the CD8^+^ T cell population Trm cells predominated over (CD69^-^CD103^-^) non-Trm CD8^+^ T cells in BA, driven by an increase in the CD103^+^CD69^+^ population (Trm cells: p=0.001, DP Trm cells: p=0.001, non-Trm CD8^+^ T cells: p=0.001) (**Figure 1C**). We did not identify differences in the Trm cell frequency comparing our control cohort to the frequency of Trm cells isolated from biopsy specimens from transplant livers prior to reperfusion from healthy pediatric liver donors (n=4 mean age 5.54 years, **Supplementary Figure 3B**).

HLA-DR^+^ myeloid cells were analyzed based on their expression of CD16 and CD14. Unbiased PhenoGraph analysis revealed an increase in CD14^+^CD16^+^ intermediate monocytes (cluster 11) (**Figure 1A**), which we could reproduce by manual gating. Further, Kupffer cells were identified as CD11b^+^CD68^+^ in the CD16^+^CD14^+^ intermediate monocytes (20, 21). Within the CD14^-^CD16^-^ fraction, we annotated a mixed population of pre-DC and pDC, based on their expression of CD123 (22), which appeared unchanged in BA (**Figure 1B**). DC2 were identified as CD1c^+^. However, this population here could also contain some recently described DC3 (23). Capsular macrophages were identified as CD14^+^CD16^-^CD68^-^CX3CR1^+^ cells (24, 25), other liver tissue macrophages also could express CD68. We further observed an increase in pro-inflammatory CD14^+^CD16^+^ monocytes, irrespective of CD68 expression (**Figures 1A, B**, **Supplementary Table 2**, clusters 11, 22 and 26).

**Figure 2:**
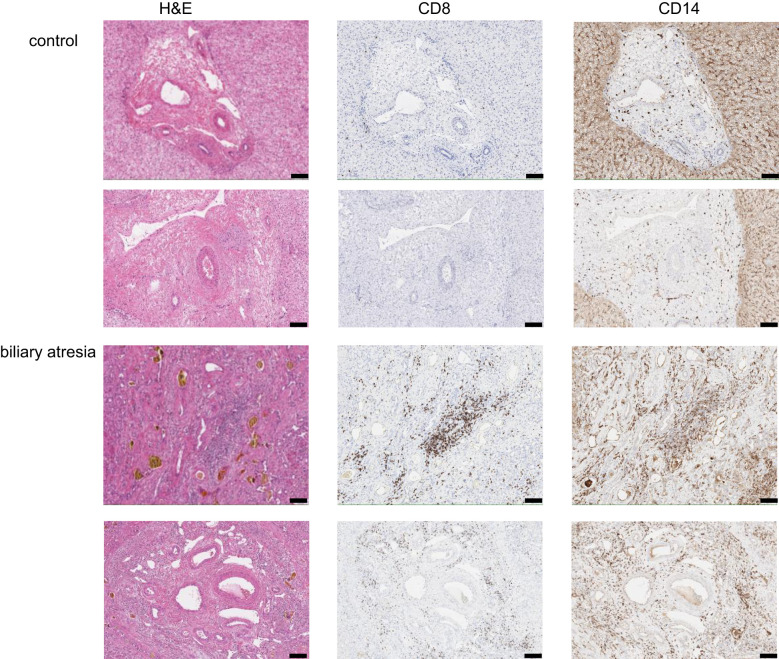
Localization of CD8^+^ T cells and CD14^+^ cells in the liver tissue. Representative images of immunohistochemical staining of portal tracts from BA and control livers (2 children per group) for H&E, CD8 and CD14. Magnification 10x, scale bar represents 100µm.

To gain an understanding of the microanatomical relationship between intrahepatic Trm and monocytes, we performed an immunohistochemical staining of representative liver tissue samples from six children per group. We found an increased frequency of CD8^+^ T cells and CD14^+^ cells around the portal tract in BA. In contrast, CD8^+^ T cells were almost completely missing from the portal areas in control livers (**Figure 2**, **Supplementary Figure 4**).

In summary, we observed an expansion of CD8^+^ Trm and CD14^+^CD16^+^ intermediate monocytes as well as a colocalization of CD8^+^ T cell and CD14^+^ monocytes in advanced BA.

### 3.3 Differential expression of functional and homing markers on CD8^+^ Trm cells in biliary atresia

The concentration of profibrogenic and proinflammatory cytokines is increased in liver cirrhosis leading to a change in the intrahepatic microenvironment (26). We hypothesized that liver Trm cells as potent producers of cytokines may be significantly influenced by or contribute to these changes. Therefore, we measured the expression of immunoregulatory and homing molecules as well as cytokine production of CD8^+^ T cell subsets in advanced BA with cirrhosis.

**Figure 3:**
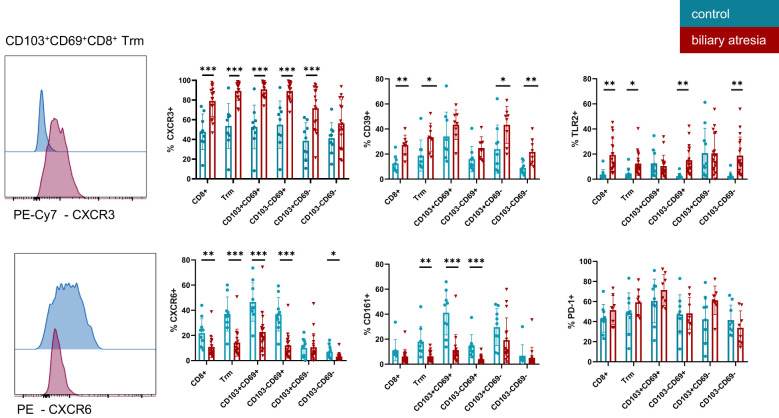
Functional characterization of tissue resident memory T cells in biliary atresia and control group. Expression of multiple markers on CD8^+^ Trm subsets, as indicated on the x-axis in BA and control group. CXCR3 (control group: n=9; biliary atresia: n=16), CD39 (control group: n=9; biliary atresia: n=8), TLR2 (control group: n=10; biliary atresia: n=18), CD161 (control group: n=9; biliary atresia: n=16), CXCR6 (control group: n=10; biliary atresia: n=18) and PD-1 (control group: n=9; biliary atresia: n=8). Data are presented as mean ± SD. *p<0.05; **p<0.01; ***p<0.001. Unpaired t-test.

The chemokine receptors CXCR3 and CXCR6 are important for tissue homeostasis and localization of intrahepatic T cells (27, 28). CD8^+^ Trm cells showed reciprocal changes in the expression of CXCR3 and CXCR6 in BA (**Figure 3**). CD8^+^ Trm cells expressed significantly higher levels of CXCR3 and lower CXCR6 in BA. This regulation appeared to be specific to Trm cells, as the expression of CXCR3 was not altered in non-Trm CD8^+^ T cells. CXCR6 expression on CD8^+^ T cells was increased in a stepwise fashion from non-Trm CD8^+^ T cells and CD103^+^CD69^-^ Trm cells through SP CD8+ Trm cells, with the highest frequency in the DP CD8^+^ Trm cell population in both groups. The frequency of CXCR6 expression was lower in the BA group compared to the control group. CX3CR1 expression was almost restricted to non-Trm CD8^+^ T cells (**Supplementary Figure 5A**).

PD-1 and CD39 are markers characterizing hepatic CD8^+^ Trm cells and expression of both markers contribute to anergy in tissues (4, 7). Expression of CD39 (E-NTPDase1—ectonucleoside triphosphate diphosphohydrolase 1) was higher on CD8^+^ Trm and non-Trm CD8^+^ T cells populations equally in BA compared to the control group. No difference was observed in the CD39 expression on DP and SP Trm in both groups. PD-1, a marker for exhausted T cells, was expressed on the majority of Trm cells, irrespective of the presence or absence of cirrhosis. Similarly, expression of the Toll-like receptor 2 (TLR2) was higher in all CD8^+^ T cells in BA-associated cirrhosis compared to the control group (**Figure 3**). CD14 expression was low in intrahepatic CD8^+^ T cells in all samples analyzed (**Supplementary Figure 5B**).

CD161, a marker for IL-17 producing cells across many lineages including the liver (29, 30), was not frequently expressed in CD8^+^ Trm cells in BA, while interestingly the DP Trm cells expressed high levels of CD161 in the control group (**Figure 3**).

We further analyzed the relative abundance of CD8^+^ T cells expressing CXCR3, CXCR6, CD161, TLR2, and CD39 within the CD3^+^ T cell compartment. Interestingly, in BA patients, not only the proportion of these marker-expressing cells within the CD8^+^ T cell subset was altered, but also their frequency within the total CD3^+^ T cell population. Specifically, we observed a higher frequency of CD8^+^ T cells expressing CXCR3, TLR2, and CD39 in BA compared to the control group, whereas the frequency of CXCR6-expressing CD8^+^ T cells was reduced. In contrast, the frequencies of CD161^+^ and CD14^+^ CD8^+^ T cells within the CD3^+^ T cell population did not differ significantly between BA patients and control children (**Supplementary Figure 5C**).

This indicates a shift of functional and tissue residency markers during BA associated cirrhosis. CD69 and CD103 expression was higher in CD8^+^ T cells representing a higher abundance of Trm cells in BA. Furthermore, CXCR3, CD39 and TLR2 expression was elevated while CD161 and CXCR6 expression was reduced in CD8^+^ T cells in BA. These phenotypic changes suggest an inflammatory and immunoregulatory shift in the liver microenvironment associated with advanced BA.

### 3.4 CD103^+^CD69^+^CD8^+^ Trm cells are the most potent cytokine producers

**Figure 4:**
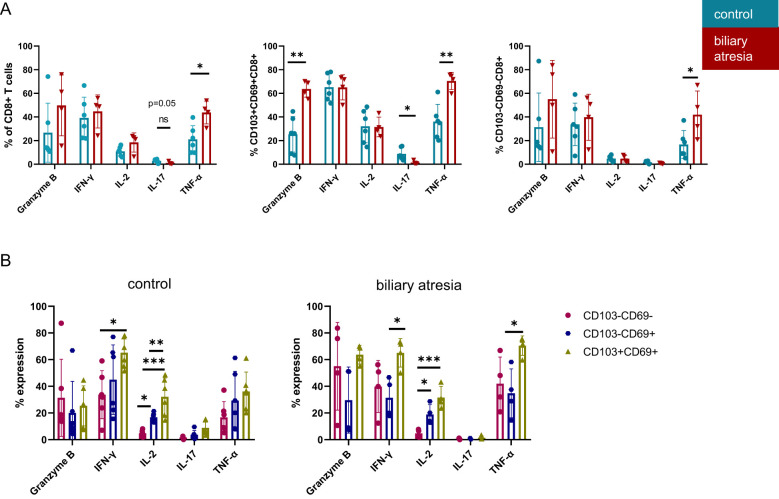
Increased cytotoxicity of CD103^+^CD69^+^CD8^+^ DP Trm cells in biliary atresia. **(A)** Cytokine production of CD8+ T cells and Trm subsets including Granzyme B, IFN-γ, IL-2, IL-17 and TNF-α in comparing BA and control group (%: control group: n=6; biliary atresia: n=4). **(B)** Cytokine production of Trm cell subpopulations. Frequencies of Granzyme B, IFN-γ, IL-2, IL-17 and TNF-α producing cells comparing CD103^-^CD69^-^CD8^+^ non-Trm CD8^+^, CD103^-^CD69^+^CD8^+^ SP Trm with CD103^+^CD69^+^CD8^+^ DP Trm populations in control group and biliary atresia (%: control group: n=6; biliary atresia: n=4). Data are presented as mean ± SD. *p<0.05; **p<0.01; ***p<0.001. **(A)** Unpaired t-test, **(B)** One-way-Anova and Tukey´s multiple comparison test.

For characterization of intrahepatic CD8^+^ T cell function, we further assessed the production of cytokines and cytotoxic mediators by intracellular staining after 6 hours of stimulation with PMA and ionomycin (**Supplementary Figure 6**). CD8^+^ T cells produced more TNF-α and IL-2 in BA (p=0.01 and p=0.08, respectively, **Figure 4A**). The increased TNF-α production was mainly driven by DP Trm cells (p=0.003), but also non-Trm CD8^+^ T cells produced increased levels of TNF-α in BA (p=0.035). IL-17 production of DP Trm cells was decreased in BA (p=0.023). Production of the serine protease granzyme B by DP Trm cells was significantly increased (p=0.002) but not significantly higher in CD8^+^ T cells (p=0.19) in BA.

Previous studies investigated the different cytokine production capability of DP Trm compared to non-Trm CD8^+^ T cells in human tissues (5, 7). As both SP and DP Trm cells were more frequent in BA, we analyzed the functional differences between these subsets in BA and the control group (**Figure 4B**). IL-2 production increased stepwise from non-Trm through SP to DP Trm cells and DP Trm cells produced the highest levels of IFN-γ in both BA and the control group (**Figure 4B**). The increased TNF-α production characteristic of DP Trm cells was specific for BA, as this was not observed in the control group.

Several of the cytokines and cytotoxic mediators produced by liver DP Trm cells in biliary atresia were also increased in the peripheral blood plasma of these patients. TNF-α (p=0.006), Granzyme B (p=0.002), and INF-γ (p=0.025) were all increased in plasma samples from children with BA, obtained immediately prior to liver transplantation (**Supplementary Figure 5D**). While this signature is consistent with the cytokine production of liver Trm cells in these patients, children with BA also showed further signs of increased systemic inflammation, such as higher CRP (**Table 2**) and IL-6 (p=0.048). Also, IL-17 not produced by hepatic CD8^+^ Trm cells in BA was increased in plasma (**Supplementary Figure 5D**) so that possibly other immune cells and other organs contributed to increased cytokine levels in plasma.

TGF-β and IL-15 initiate the development of a liver-Trm phenotype in CD8^+^ T cells (7, 31). Interestingly, we found TGF-β, but not IL-15, (p=0.004 and p=0.4, respectively) significantly increased in the plasma of children with BA (**Supplementary Figure 4D**).

CD8^+^ Trm cells in advanced BA showed a distinct phenotype compared to their counterparts in the control livers. The differential regulation of chemokine receptors, pro- and anti-inflammatory mediators indicates a shift in the overall function of CD8^+^ Trm cells. While CD8^+^ Trm cells in control livers expressed CD161 and CXCR6 as well as produced more IL-17, Trm cells in BA expressed CXCR3, TLR2 and CD39, increased levels of CD69 and CD103 and were more likely to produce TNF-α and Granzyme B, which also correlate with cytokine composition in patient plasma.

### 3.5 Monocytes induce a proinflammatory Trm-like phenotype in a cell-contact dependent manner *in vitro*

**Figure 5:**
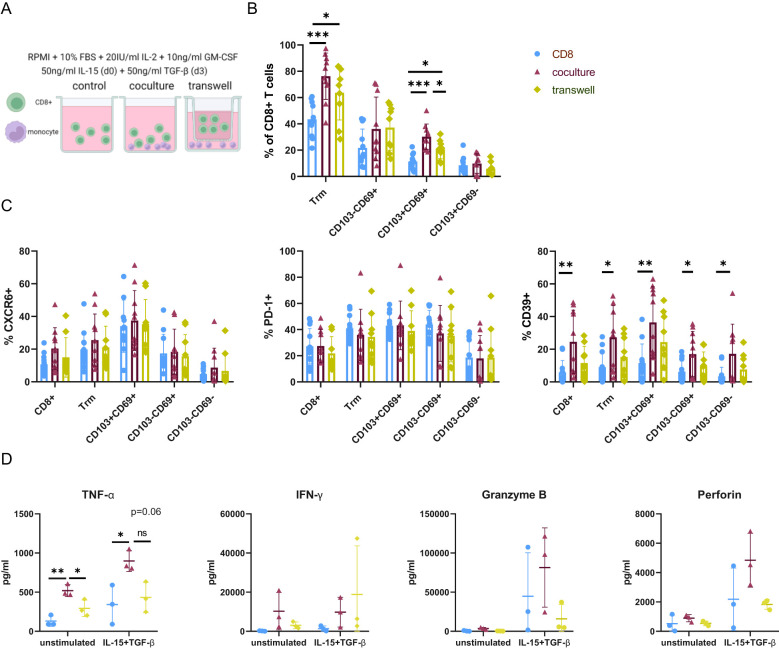
Monocytes induce a proinflammatory Trm-like phenotype in a cell-contact dependent manner *in vitro.* **(A)** Schematic representing culture setup. CD8^+^ T cells were cultured alone (control) or with autologous monocytes where cell-cell contact was allowed (coculture) or prevented (transwell). IL-15 was supplemented to the medium on day 0 and TGF-β added on day 3 to the medium containing 20U/ml IL-2 and 10ng/ml GM-CSF. Analysis was carried out on day 7. **(B)** Frequencies of CD103 and CD69 expressing CD8+ T cells in the different culture setups. **(C)** Frequencies of CXCR6, CD39, PD-1 expression in each Trm-like cell population in the different cell culture setups. **(D)** Concentration cytokines and cytotoxic mediators was measured in the cell culture supernatant by cytokine bead array. Comparison of TNF-α, IFN-γ, Granzyme B, and Perforin concentration between the control and the Trm-induction medium, as well as between different culture setups. One of three representative experiments is shown. Legend for colors and symbols representing different cell culture setups for Figures B–D is indicated in the upper right corner. Data are presented as mean ± SD. *p< 0.05; **p< 0.01; ***p< 0.001; ns, not significant. **(B–D)** One-way-Anova and Tukey´s multiple comparison test. **(A)** Created with Biorender.com.

We found a higher frequency of both CD8^+^ Trm and CD14^+^CD16^+^ proinflammatory monocytes in advanced biliary atresia compared to the control group (**Figure 1**). Furthermore, CD8^+^ and CD14^+^ cells were both identified in portal areas in BA (**Figure 2**). We investigated whether pro-inflammatory monocytes could reinforce a liver Trm-like phenotype in autologous T cells *in vitro*. We induced a proinflammatory phenotype on monocytes purified from healthy adult blood and expanded with GM-CSF (32). Autologous CD8^+^ T cells were cultured alone (control) or added to the monocyte cultures either separated by a semipermeable membrane allowing for the exchange of paracrine mediators but no cell-cell contacts (transwell) or mixed in culture allowing for both paracrine and physical interactions (coculture). We used a previously described protocol mimicking the cytokine milieu of the liver to facilitate a liver-Trm-like phenotype in peripheral blood CD8^+^ T cells, by adding IL-2 and IL-15 to the initial medium followed by TGF-β on day three (**Figure 5A**) (7). Since a role of antigen-mediated immune activation has not been identified in biliary atresia, we did not use TCR-mediated stimulation in this model.

On day seven, the frequency of cells with a Trm-like phenotype was significantly increased in the presence of monocytes, and this occurred independently of cell-cell contact. This change was mainly attributed to DP Trm-like cells, as no significant differences between culture setups could be observed for SP Trm-like cells. In the coculture setup, DP Trm cells were significantly increased compared to both control and transwell, and in the transwell setup significantly increased compared to the control (**Figure 5B**).

CD39 expression, which was higher in CD8^+^ T cells in BA (**Figure 3**), was also increased *in vitro* in the coculture setup. This increase was not dependent on the upregulation of Trm markers CD103 and CD69. No cell-contact-induced upregulation of PD-1 and CXCR6 was observed (**Figure 5C**). The importance of physical cell-cell interactions for Trm cell induction and phenotype was further emphasized by an increase of CD69 and CD39 expression of CD8^+^ T cells in coculture in the control medium without Trm-inducing cytokines IL-15 or TGF-β (**Supplementary Figures 7A, B**).

In parallel, we analyzed the concentrations of cytokines and cytotoxic mediators in the cell culture supernatant. In the coculture, TNF-α concentration in the supernatant was enriched compared to the control and the transwell condition independent of Trm inducing cytokines and also mimicking *ex vivo* changes in pediatric liver cirrhosis. Even though not significant, IFN-γ, Granzyme B and Perforin were slightly increased in the coculture setup upon addition of IL-15 and TGF-β (**Figure 5D**).

## 4 Discussion

Cholestatic diseases in infancy, especially biliary atresia, are the leading causes of end-stage liver cirrhosis necessitating liver transplantation early in life^4^. The immunologic mechanisms leading to or contributing to liver cirrhosis, end-stage liver failure and the need for liver transplantation are poorly understood. Here, we presented an in-depth characterization of intrahepatic CD8^+^ T cell and monocyte populations in an infant cohort of 18 patients with biliary atresia and 10 age-matched controls. We elucidate a possible role of cytotoxic CD8^+^ Trm cells in the pathogenesis of biliary atresia. Furthermore, we described important changes in innate immune subsets in biliary atresia, most importantly an increase in proinflammatory monocytes. We provided an *in vitro* model showing proinflammatory monocytes can reinforce the Trm phenotype similar to BA livers.

We identified a relative increase of tissue resident CD8^+^ T cell populations paralleled by a decrease of CD103^-^CD69^-^ non-Trm CD8^+^ T cells within the hepatic CD8^+^ T cell pool in advanced BA. In particular, the CD103^+^CD69^+^ DP population increased most prominently, while CD103^-^CD69^-^ non-Trm CD8^+^ T cells frequencies remained relatively constant. The expression of CD69 alone is sufficient for tissue retention as it results in a decreased expression of S1PR1, which facilitates the egress from the tissue into the circulation (5). CD103 interacts with E-cadherin on hepatocytes and could therefore further maintain tissue residency (10). The frequency of CD8^+^ Trm cells, particularly CD103^+^ Trm cells, has been shown to be higher in inflammatory liver diseases and was accompanied by changes in phenotype and functional profiles (7, 10, 11, 33, 34).

We showed an increase in CXCR3 and a decrease in CXCR6 expression in CD8^+^ T cells in BA. Both chemokine receptors CXCR3 and CXCR6 modulate T cell maintenance in the liver and guide these cells to their respective homing sites (27, 28, 35). An increase of CXCR3^+^CD8^+^ T cells and a localization around the portal tract was described in biliary atresia and adult primary biliary cholangitis (36, 37), while CXCR6^+^ cells are directed to the cholangiocytes by their respective ligand CXCL16 (27, 38). CXCR3^+^ cells possess increased transendothelial migratory capacity and could be recruited from the periphery (39). CXCR3 is also crucial for liver residency as blocking of CXCR3 prevented tissue accumulation in mice (9, 35). Consequently, this could indicate, as the frequency of CXCR6^+^CD8^+^ T cells decreased in the CD3^+^ T cell population, that CXCR3^+^CD8^+^ T cells may represent newly migrating cells responsible for the pathological increase of CD8^+^ Trm cells seen in our study in BA. In line with the literature, the expression of CX3CR1 was restricted to non-Trm CD8+ T cells. In agreement with previous data CX3CR1 could serve as a negative marker for Trm cells (9, 18, 21).

We saw an increased expression of CD39 and TLR2 in BA in CD8+ T cells that was not restricted to CD8^+^ Trm cells, even though the frequency of CD39 was highest in DP Trm cells. TLR2 has been reported to reduce the threshold of activation on CD8^+^ T cells and is a suggestive of increased metabolic activity (40, 41). Further, increased TLR2 expression on intrahepatic CD8^+^ T cells may be indicative of cell-cell contacts between monocytes and CD8^+^ T cells involving membrane transfer as suggested in a recent publication by Pallet et al (42). However, in our cohort we could not identify high co-expression of CD14^+^ and CD8^+^ on T cells in either study group, nor in liver samples from two adult organ donors.

CD8^+^ Trm cells react swiftly upon reactivation (5, 7, 43). In fact, these rapid local immune memory responses might represent the main function of tissue resident memory T cells (4). Some previous studies addressed antigen-specific induction and activation of liver Trm cells in humans, as well as their possible role in antimicrobial defense (7, 44). In contrast to these previously addressed clinical situations, our pediatric cohort represents a somewhat unique setting in that an underlying antigen leading to generation of liver Trm cells cannot clearly be identified, even though oligoclonality of T cells in BA livers has been shown in one study (15). Upon unspecific stimulation and intracellular staining, CD8^+^ Trm cells from cirrhotic infant livers in our cohort showed most prominently an increased production of proinflammatory TNF-α and Granzyme B by DP Trm cells. In BA, DP Trm cells produced the highest frequency of TNF-α, with no differences in the control group, indicating that the increased TNF-α production might represent a Trm phenotype specific to advanced BA. Granzyme B producing cytotoxic CD103^+^CD69^+^CD8^+^ Trm cells could contribute to liver damage in autoimmune hepatitis (AIH) (10).

On the other hand, both the expression of CD161 and of IL-17 in DP Trm cells was restricted to the control group and absent in BA, representing a possible loss of some antimicrobial immune-surveillance functions (even though IFN-γ production was preserved). There is some evidence suggesting a pathogenic role of IL-17 in biliary atresia (14, 17). However, CD8^+^ Trm do not appear to be an important contributor, also not in our cohort.

The higher relative frequency and increased cytotoxic capacity of Trm cells in BA may also contribute to tissue destruction and fibrosis in the liver. Increased presence of proinflammatory TNF-α in liver cirrhosis is well documented (26, 45). TNF-α activates hepatic stellate cells, the most prominent cells in liver fibrogenesis, as well as promotes their survival. Thus, TNF-α takes an important role in the progression and perpetuation of liver fibrosis (45). Active HSC, Kupffer cells, CD14+CD16+ monocytes and hepatocytes produce TGF-β, a cytokine directly involved in tissue remodeling and scarring, which is also released by apoptotic cells (26). Importantly, we showed both CD14+CD16+ monocytes and Kupffer cells to be increased in biliary atresia. Fitting this, we measured increased plasma levels of both TGF-β and TNF-α in children with BA, and similar observations were made in an adult cirrhosis cohort (46–48). Indeed, a possible benefit of blocking TNF-α with infliximab to prevent liver tissue destruction has been shown in an adult cohort of patients with autoimmune hepatitis (49).

In our cohort, we saw an increase in tissue proinflammatory intermediate monocytes (CD16^+^CD14^+^) in BA compared to the control group. CD14^+^CD16^+^ intermediate monocytes were shown to contribute to liver cirrhosis in adults (48, 50, 51). Histologic stainings in an earlier study suggested an increase of CD14^+^ monocytes in children with cholestatic diseases (52). Humoral cues from the liver microenvironment may induce Trm differentiation in transitory CD8^+^ T cells, and possible paracrine mediators, such as IL-15 and TGF-β, have been described previously (7, 31). Especially the concentration of TGF-β spikes in cirrhosis (26). The liver represents a cell-rich microenvironment, and thus physical cell-cell interactions may also play a role in the regulation of Trm development. In our *in vitro* model, cell-cell contacts between monocytes and autologous CD8^+^ T cells without antigen led to the induction of a Trm-like phenotype and to an upregulation of TNF-α and CD39, but not of PD-1 similar to what is seen in advanced BA. A recent study demonstrated that Kupffer cells have the capacity to present antigens to CD8^+^ T cells inducing differentiation of CD8^+^ T cells into functional effector cells in an IL-2 dependent manner, while hepatocyte mediated priming led to dysfunctional CD8^+^ T cells (53, 54). Monocytes are known to be capable of trans-presenting membrane-bound IL-15 and TGF-β and thereby contributing to CD103 expression (55, 56). Membrane-bound TGF-β on monocytes presented directly to CD8^+^ T cell could thereby enhance CD103 expression. Furthermore, Trm cells in the liver express LFA-1, facilitating their extravasation through adhesion to ICAM-1, which is expressed by monocytes, endothelial cells as well as hepatocytes and Trm cell themselves (21, 57, 58).

Our study has several limitations. Our cohort of 28 liver transplanted children represents a unique chance to study the pediatric hepatic immune system. However, as most studies are done in adult organs, we can only compare our data with results obtained by the analysis of adult Trm cells. Adult Trm cells are transcriptionally different as a recently published study by Connor et al. highlights (59). The control group consisted of ten children lacking signs of liver damage, but who themselves were affected by various genetic metabolic diseases that might each have unknown immunologic phenotypes. No difference in Trm cell frequency was observed between our control cohort and biopsy specimens taken from transplant livers of healthy pediatric liver donors prior to reperfusion and the heterogeneity of diseases in the control group should balance out singular immunopathologic changes in the underlying diseases. Of note, reduced T cell receptor engagement of T cells from patients with glycogen storage disease type 1b has been reported, which is why we did not include patients with this disease in stimulation assays (60). Children in the BA group had very advanced disease with end-stage cirrhosis as the only time point of analysis was at liver transplantation. Thus, the immunopathologic changes might not always be representative of the early phase of liver destruction, which is where potential therapeutic interventions could have the most benefit. Finally, the experimental groups were too small to evaluate any age-related effects that might significantly influence immunologic phenotypes.

In summary, we propose that for the induction of a Trm-like phenotype in CD8^+^ T cells, cellular as well as humoral cues from the liver microenvironment might play an important role. We describe parallel increases involving pro-inflammatory CD16^+^ monocytes and CD103^+^CD69^+^CD8^+^ Trm cells in children with advanced biliary atresia. Histologically, monocytes and CD8^+^ T cells colocalized near portal tracts in cirrhotic liver tissue. Furthermore, the previously described effect of sequential IL-15 and TGF-β exposure *in vitro* could be further enhanced by coculture of T cells with monocytes in a contact-dependent manner. In BA, Trm adopted a cytotoxic rather than antimicrobial phenotype and thus may contribute to the immunopathology of irreversible liver destruction, however regulatory mechanisms such as an increased CD39 expression may be in place to counteract immune activation. Therapies targeting Trm cells are already in clinical use in multiple sclerosis and are evaluated for inflammatory bowel disease and other autoimmune diseases (61, 62). These novel drugs, as well as biologicals targeting the TNF-a signaling pathway may in the future find a role in slowing the progression to liver cirrhosis in BA and prolonging transplant free survival.

## Data Availability

The raw data supporting the conclusions of this article will be made available by the authors, without undue reservation.
