# Supplementary material for: Expansion of CD103+CD69+CD8+ cytotoxic liver tissue resident memory T cells and inflammatory monocytes in advanced biliary atresia
Source: Front Immunol. 2025 Jun 18;16:1567645. doi: 10.3389/fimmu.2025.1567645 (PMC12213756; doi:10.3389/fimmu.2025.1567645)
Supplement: Supplementary file 1 [file DataSheet1.docx]

Supplementary Material

Sibbertsen et al: Expansion of CD103^+^CD69^+^CD8^+^ cytotoxic liver tissue resident memory T cells and inflammatory monocytes in advanced biliary atresia

# Supplementary Figures

**
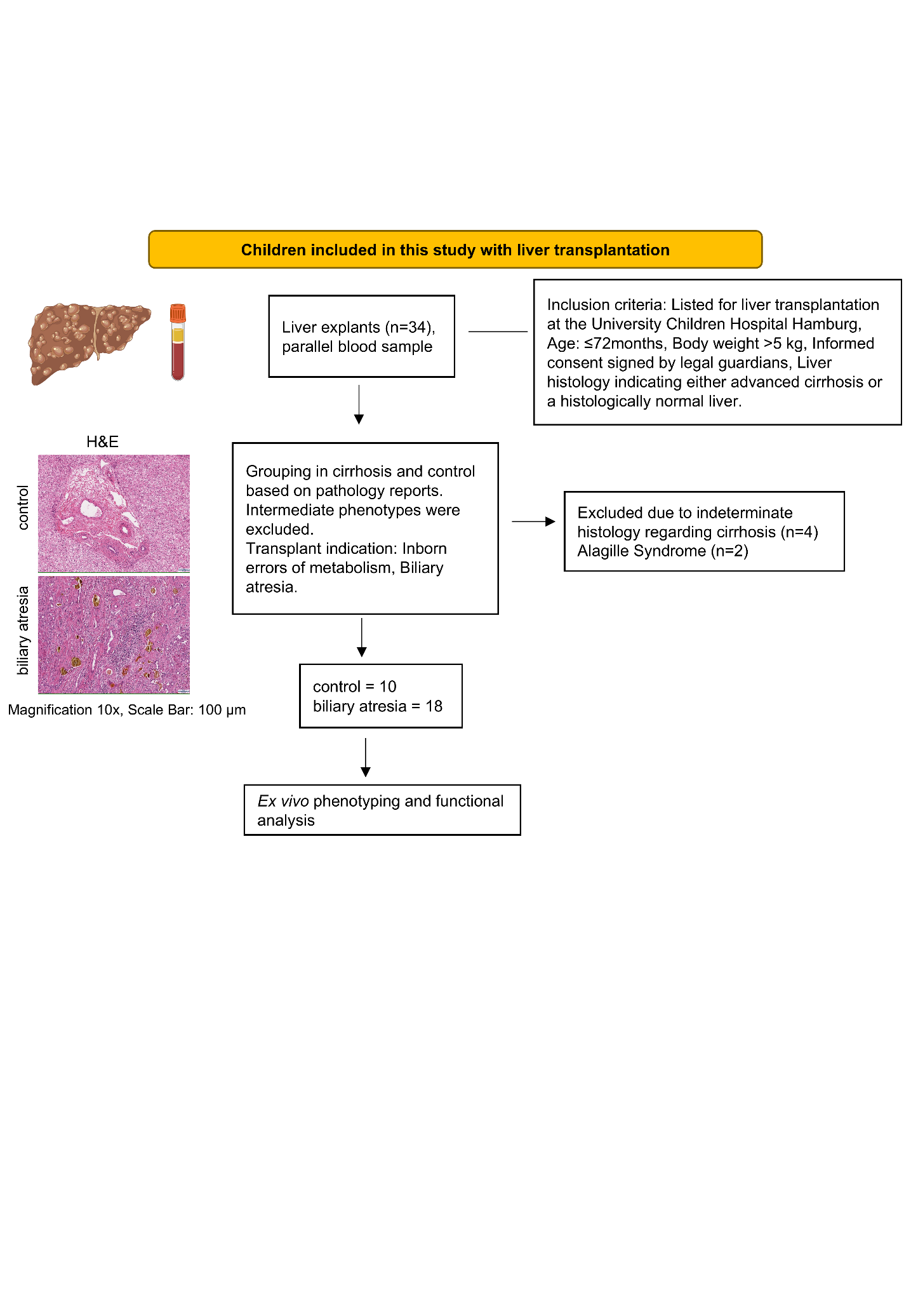
**

**Supplementary Figure 1.** Schematic representation of the study design.

**
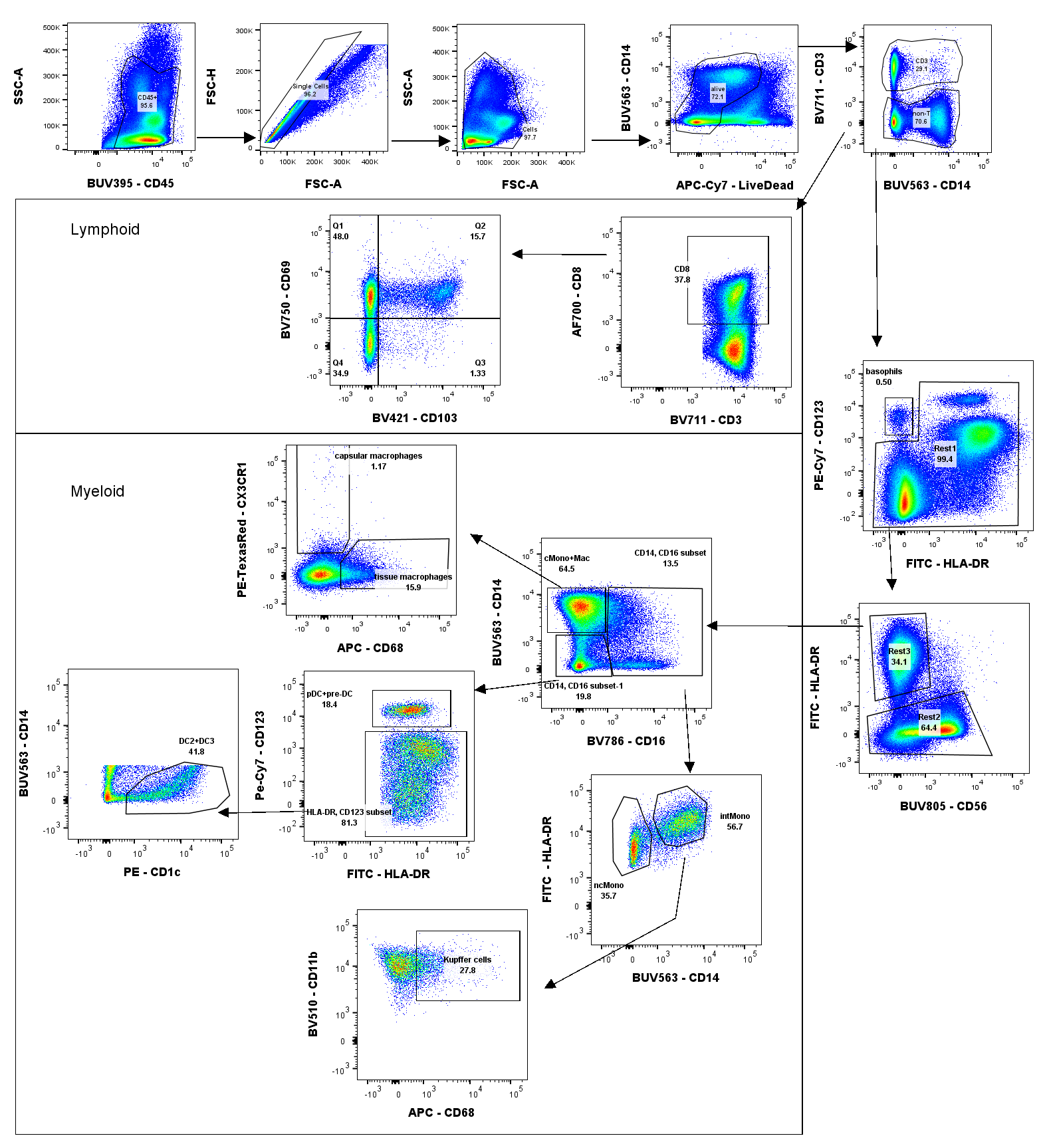
**

**Supplementary Figure 2.** Sequential gating strategy to identify cell populations of interest as indicated in the figure


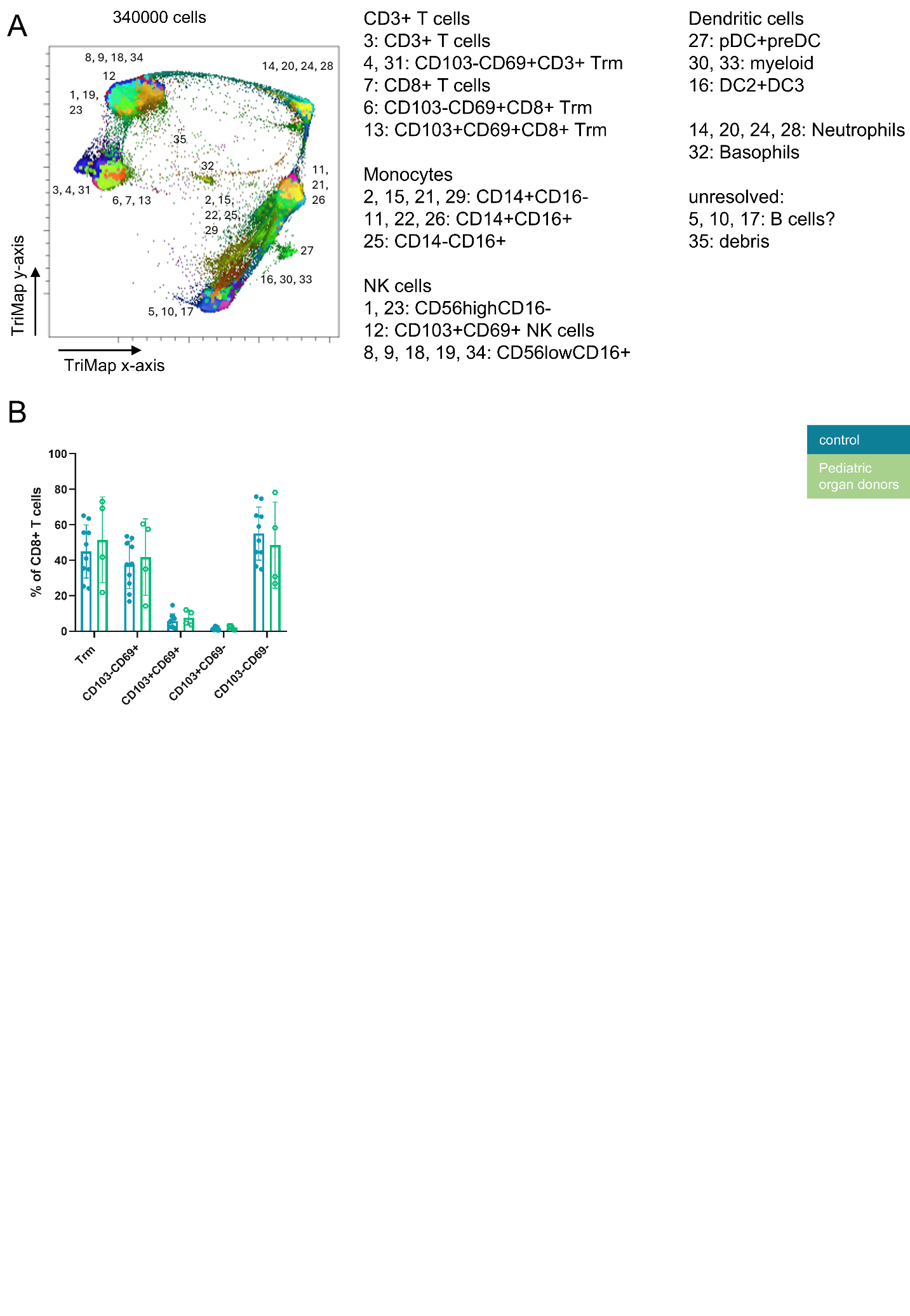


**Supplementary Figure 3.** (A) Equivalent number of CD45^+^ live cells were concatenated, visualized with the FlowJo Plugin TriMap using default settings and clustered using PhenoGraph, with k set to 20. Each cluster was annotated based on the expression of linage markers. The annotation is shown next to the figure (control group: n=10; biliary atresia: n=17). (B) Frequencies of CD8^+^ Trm cells and subpopulations in CD8^+^ T cells (control group: 10 (mean age: <1 year); pediatric organ donors: n=4 (mean age: 5.45 years)). Data are presented as mean ± SD.


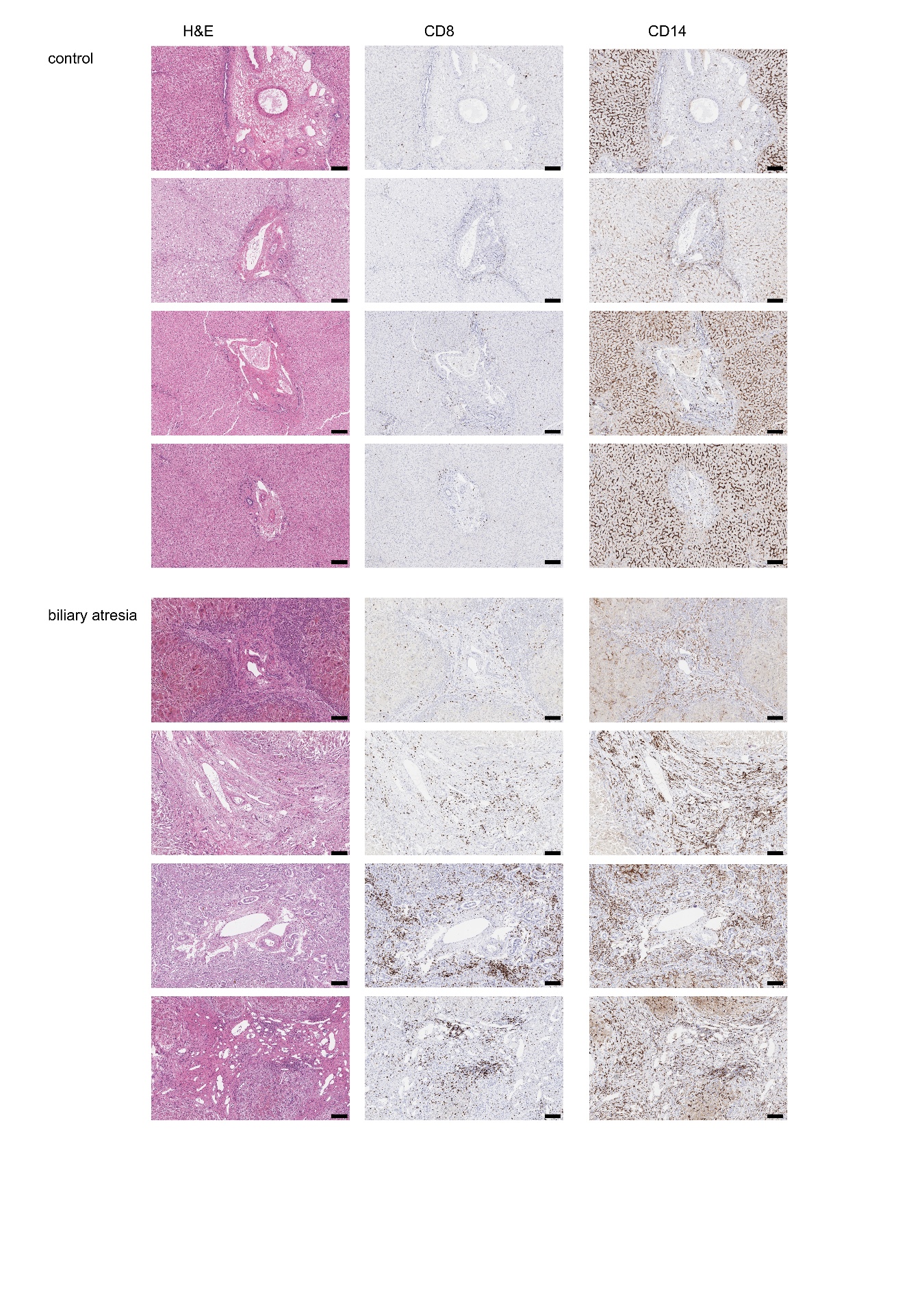


**Supplementary Figure 4.** Localization of CD8^+^ T cells and CD14^+^ cells in the liver tissue. Representative images of immunohistochemical staining of portal tracts from BA and control livers (4 additional children per group) for H&E, CD8 and CD14. Magnification 10x, scale bar represents 100µm.


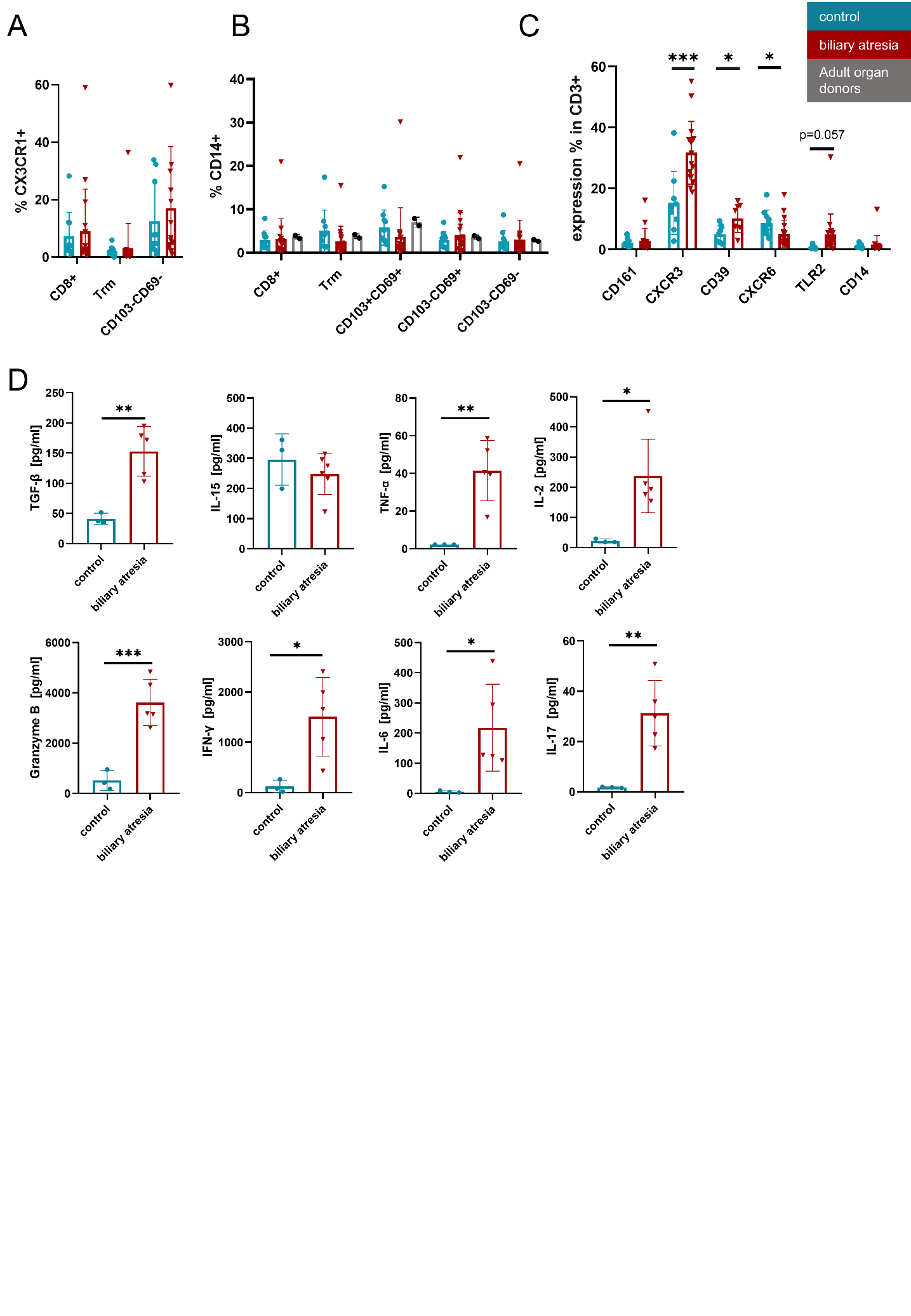


**Supplementary Figure 5.** (A) Frequencies of CX3CR1 positive cells in CD8^+^, Trm and conventional T cells (%: control group: n=10; cirrhosis biliary atresia n=17). (B) Frequencies of CD14^+^ cells in CD8^+^ T and CD8^+^ Trm subsets and non Trm CD8^+^ T cells (control children: n=10; biliary atresia: n=18, adult organ donors: n=2). (C) Frequencies of CD161^+^CD8^+^, CXCR3^+^CD8^+^, CD39^+^CD8^+^, CXCR6^+^CD8^+^ and TLR2^+^CD8^+^ T cells in CD3^+^ T cells in cirrhosis compared to control.  (D) Concentration TGF-b, IL-15, TNF-α, IL-2, Granzyme B, IFN-γ and IL-17 in plasma of children just before liver transplantation, as measured by cytokine bead array (control group: n=3; biliary atresia: n=5). Data are presented as mean ± SD. *p<0.05; **p<0.01; ***p<0.001. (A-D) Unpaired t-test.

**
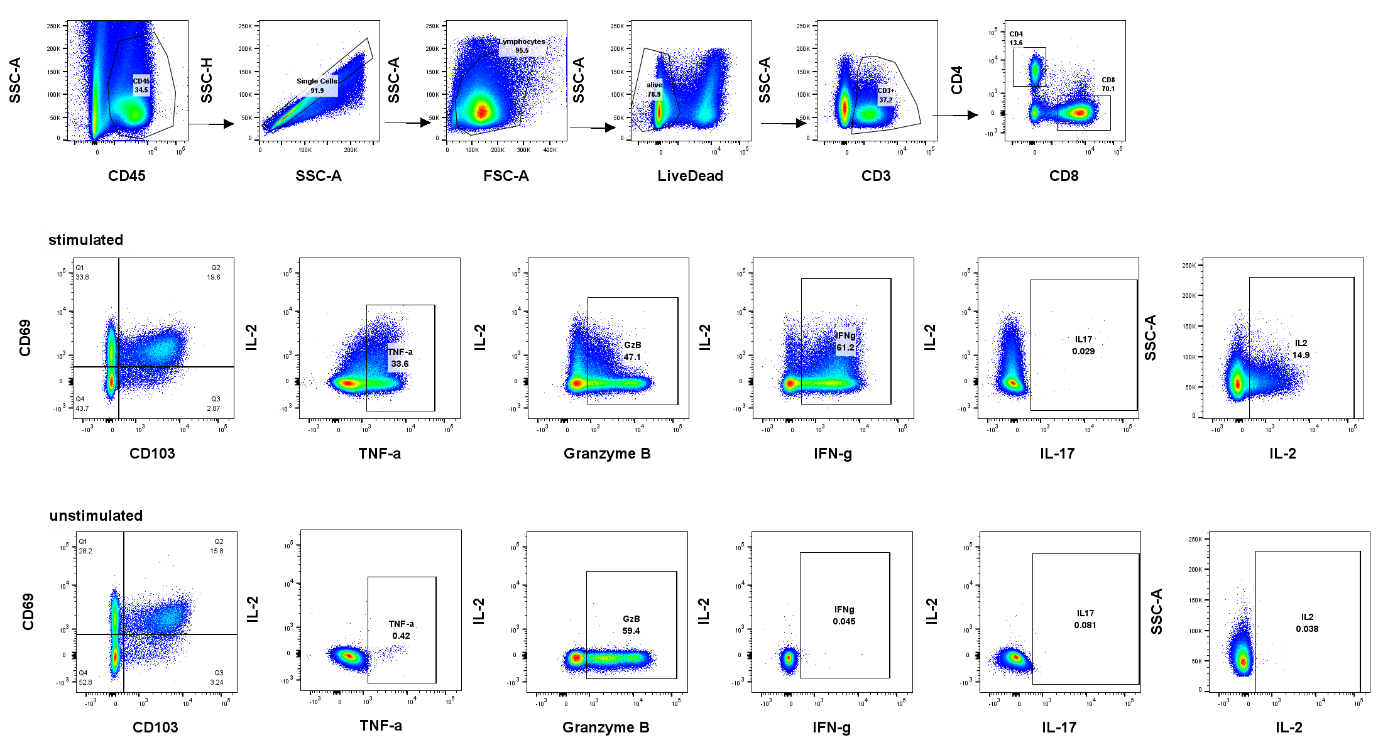
Supplementary Figure 6.** Sequential gating strategy for intracellular staining shown on a representative sample (stimulated and unstimulated). Cytokines and cytotoxic mediators were gated in the CD8^+^ T cell population.

**
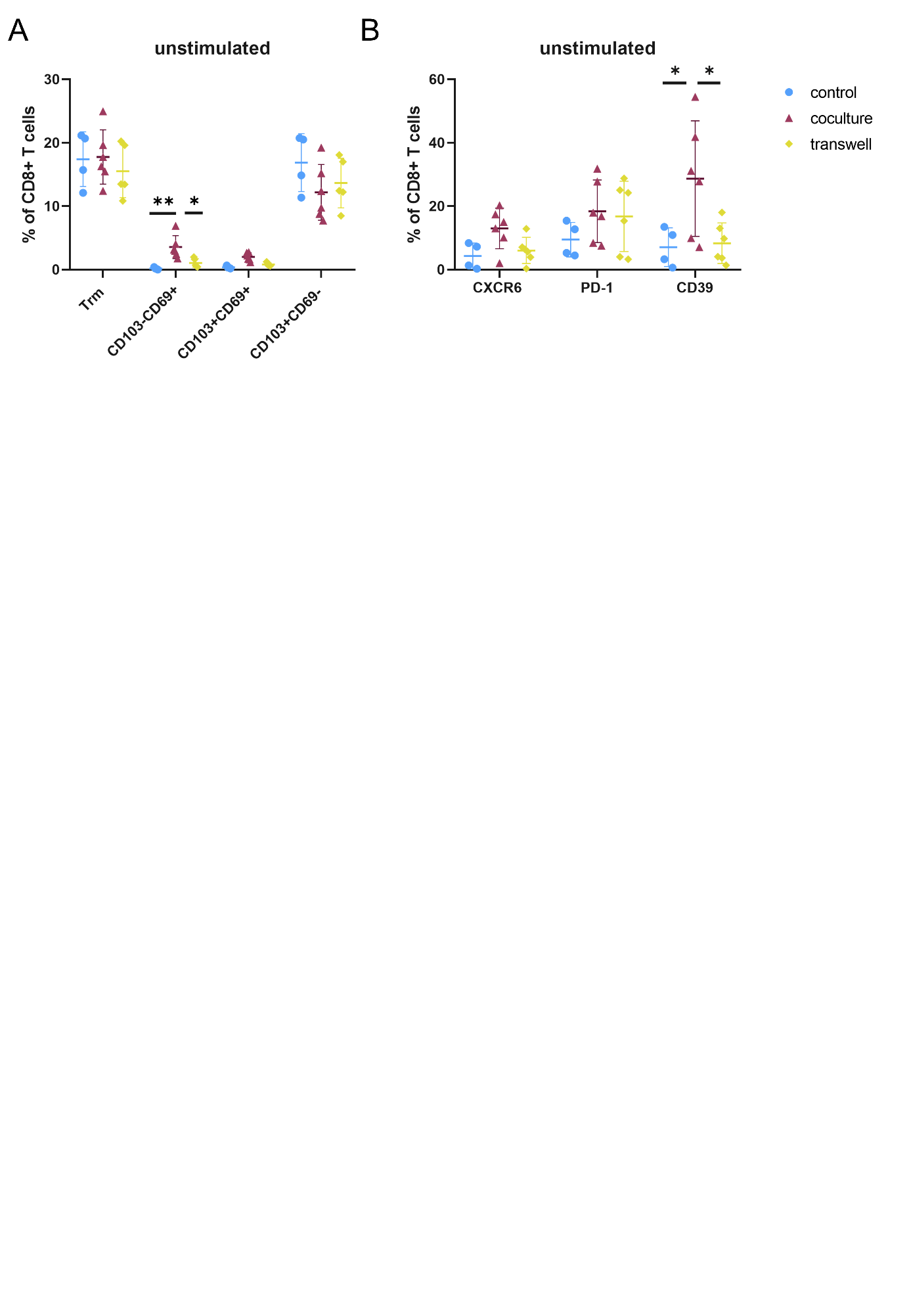
**

**Supplementary Figure 7.** CD8^+^ T cells were cultured alone (control) or with autologous monocytes where cell-cell contact was allowed (coculture) or prevented (transwell). The control medium contained 20U/ml IL-2 and 10ng/ml GM-CSF. The expression of CD103, CD69 (A), CXCR6, PD-1 and CD39 (B) in the CD8^+^ T cell population was analyzed on day 7 of the experiment. Legend for colors and symbols representing different cell culture setups for is indicated in the upper right corner. Data are presented as mean ± SD. *p< 0.05; **p< 0.01; ***p< 0.001; ns – not significant. One-way-Anova and Tukey´s multiple comparison.

# Supplementary Tables

## Supplementary Table 1: Antibodies for flow cytometry staining

| Fluorophore | Target | Clone | Supplier |
| --- | --- | --- | --- |
| AF700 | CD8 | RPA-T8 | Biolegend |
| APC | CD68 | Y1/82A | Biolegend |
| APC | γδT | 11F2 | Milteny Biotec |
| APC | GLUT1 | 202915 | BD Bioscience |
| APC | IFN-γ | 4S.B3 | Biolegend |
| APC-Cy7 | LiveDead |  | Milteny Biotec |
| BUV395 | CCR6 | 11A9 | BD Bioscience |
| BUV395 | CD4 | SK-3 | BD Bioscience |
| BUV395 | CD45 | HI30 | BD Bioscience |
| BUV496 | CD39 | TU66 | BD Bioscience |
| BUV563 | CD14 | M5E2 | BD Bioscience |
| BUV563 | CD8 | RPA-T8 | BD Bioscience |
| BUV661 | HLA-DR | G46-6 | BD Bioscience |
| BUV805 | CD56 | B159 | BD Bioscience |
| BV421 | CD103 | Ber-ACT8 | Biolegend |
| BV510 | CD11b | ICRF44 | Biolegend |
| BV510 | CD45RA | HI199 | Biolegend |
| BV510 | CD8 | SK1 | Biolegend |
| BV510 | Ki67 | Ki-67 | Biolegend |
| BV605 | CD161 | HP-3G10 | Biolegend |
| BV605 | CD36 | CB38 | BD Bioscience |
| BV650 | CD27 | O323 | Biolegend |
| BV650 | CD98/SLC3A2 | UM7F8 | BD Bioscience |
| BV711 | CD3 | OKT3_ | Biolegend |
| BV711 | CD4 | OKT4_ | Biolegend |
| BV711 | CD4 | SK1 | Biolegend |
| BV737 | CD32 | 3D3 | Biolegend |
| BV750 | CD69 | FN50 | Biolegend |
| BV786 | CD16 | 3G8 | Biolegend |
| BV786 | CD3 | OKT3_ | Biolegend |
| FITC | CD28 | CD28.2 | Biolegend |
| FITC | Granzyme B | QA18A28 | Biolegend |
| FITC | HLA-DR | G46-6 | BD Bioscience |
| FITC | TLR2 | W15145C | Biolegend |
| PE | CD1c | L161 | Biolegend |
| PE | CXCR6 | K041E5 | Biolegend |
| PE | IL-17 | BL168 | Biolegend |
| PE | TCR Vα7.2 | 3C10 | Biolegend |
| PE-CF594 | CX3CR1 | 2A9-1 | BD Bioscience |
| PE-Cy7 | CD123 | 6H6 | Biolegend |
| PE-Cy7 | CD39 | A1 | Biolegend |
| PE-Cy7 | CD71 | CY1G4 | Biolegend |
| PE-Cy7 | CXCR3 | G025H7 | Biolegend |
| PE-Dazzle | CD45 | HI30 | Biolegend |
| PE-Dazzle | IL-2 | MQ1-17H12 | Biolegend |
| PerCP-Cy5.5 | TNF-α | MAb11 | Biolegend |

## Supplementary Table 2:

Annotation of each cluster based on the expression of linage and functional markers and statistical analysis with students t-test for the PhenoGraph Analysis comparing control with BA.

| cluster | annotation | phenotype | p-value | Mean in control | Mean in BA |
| --- | --- | --- | --- | --- | --- |
| 1 | NK cells | CD56^+^CD16^-^ | <0.001 | 18.25 | 5.35 |
| 2 | CD14^+^CD16^-^ | CD39^+^CD32^+^CD11b^+^CD36^high^CD98^+^ | 0.301 | 7.52 | 11.09 |
| 3 | CD3^+^ T cells |  | 0.116 | 8.12 | 5.07 |
| 4 | CD3^+^ T cells | CD103^-^CD69^+^ | 0.206 | 4.90 | 7.48 |
| 5 | unknown |  | 0.250 | 6.27 | 4.32 |
| 6 | CD8^+^ T cell | CD103^-^CD69^+^ | 0.011 | 2.66 | 7.49 |
| 7 | CD8^+^ T cell |  | 0.214 | 3.78 | 5.95 |
| 8 | NK cells | CD56^+^CD16^+^ | 0.181 | 5.28 | 3.98 |
| 9 | NK cells | CD56^+^CD16^+^ | 0.184 | 3.66 | 5.39 |
| 10 | unknown |  | 0.007 | 7.08 | 1.51 |
| 11 | CD14^+^CD16^low^ | CD39^+^CD32^+^CD11b^+^CD36^+^CD98^+^ | <0.001 | 0.30 | 8.17 |
| 12 | NK cells | CD56^+^CD16^-^CD103^+^ | 0.001 | 0.74 | 6.06 |
| 13 | CD8^+^ T cells | CD103^+^CD69^+^CD98^+^ | <0.001 | 0.76 | 5.69 |
| 14 | Neutrophils | CD11b^+^CD16^+^ | 0.569 | 2.12 | 3.31 |
| 15 | CD14^+^CD16^-^ | CD11b^+^CD36^high^CD98^+^ | 0.943 | 2.70 | 2.56 |
| 16 | DC2+DC3 | CD39^+^CD32^+^CD36^low^CD1c^+^ | 0.009 | 3.53 | 1.16 |
| 17 | unknown |  | 0.092 | 4.03 | 0.62 |
| 18 | NK cells | CD56^+^CD16^+^ | 0.983 | 2.13 | 2.15 |
| 19 | NK cells | CD56^+^CD16^+^ | 0.799 | 1.91 | 2.14 |
| 20 | Neutrophils | CD11b^+^CD16^+^ | 0.979 | 2.00 | 2.03 |
| 21 | CD14^+^CD16^-^ | CD68^+^CD39^+^CD32^+^CD11b^+^CD36^high^CD98^+^ | 0.173 | 3.44 | 0.09 |
| 22 | CD14^+^CD16^+^ | CD39^+^CD32^+^CD11b^+^CD36^+^CD98^+^ | 0.875 | 1.47 | 1.31 |
| 23 | NK cells | CD56^+^CD16^+-^ | 0.202 | 1.52 | 1.12 |
| 24 | Neutrophils | CD11b^+^CD16^+^ | 0.144 | 1.39 | 0.40 |
| 25 | CD14^+^CD16^+^ | CD39^+^CD32^+^CD11b^low^CD36^low^CD98^low^ | 0.075 | 0.48 | 1.18 |
| 26 | CD14^-^CD16^+^ | CD32^+^CD11b^+^CD36^+^CD98^+^ | 0.451 | 0.01 | 1.64 |
| 27 | preDC+pDC | Cd36^low^CD98^high^CD123^+^ | 0.374 | 0.81 | 0.61 |
| 28 | Neutrophils | CD11b^+^ | 0.288 | 0.38 | 0.81 |
| 29 | CD14^+^CD16^-^ | CD32^+^CD11b^+^CD36^high^CD98^+^ | 0.195 | 0.95 | 0.04 |
| 30 | Myeloid* | CD39^+^CD98^low^ | 0.040 | 0.71 | 0.11 |
| 31 | CD3^+^ T cells | CD39^+^CD103^-^CD69^low^CD98^+^ | 0.029 | 0.16 | 0.54 |
| 32 | Basophils | CD32^+^CD11b^low^CD123^+^ | 0.563 | 0.35 | 0.29 |
| 33 | Myeloid* | CD39^+^CD98^+^ | 0.095 | 0.27 | 0.15 |
| 34 | NK cells | CD56^+^CD16^+^ | 0.078 | 0.18 | 0.11 |
| 35 | debris |  | 0.396 | 0.17 | 0.12 |

*Clusters 30 and 33 represent most likely CD14^-^CD141^+^ DC1 cells or CD14^-^CD5^-^CD163^+^ DC3 cells described by Dutertre et al. ^1^. We also confirmed that these populations have a high expression of CD98 (data not shown).

Literature

1. Dutertre, C. A. *et al.* Single-Cell Analysis of Human Mononuclear Phagocytes Reveals Subset-Defining Markers and Identifies Circulating Inflammatory Dendritic Cells. *Immunity* **51**, 573-589.e8 (2019).
